# Serial 7 T MRI demonstrates reversible hippocampal T2 abnormalities in transient global amnesia

**DOI:** 10.3389/fneur.2026.1875469

**Published:** 2026-07-30

**Authors:** Yoshitaka Ishiguro, Naoto Jingami, Tomohisa Okada, Sachi Okuchi, Yasutaka Fushimi, Yuji Nakamoto, Ryosuke Takahashi, Riki Matsumoto, Shigeru Ohtsuru

**Affiliations:** 1 Department of Primary Care and Emergency Medicine, Kyoto University Graduate School of Medicine, Kyoto, Japan; 2 Department of Neurology, Kyoto University Graduate School of Medicine, Kyoto, Japan; 3 Human Brain Research Center, Kyoto University Graduate School of Medicine, Kyoto, Japan; 4 Department of Diagnostic Imaging and Nuclear Medicine, Kyoto University Graduate School of Medicine, Kyoto, Japan

**Keywords:** 7-tesla MRI, CA1, diffusion-weighted imaging, hippocampus, reversibility, T2-weighted imaging, transient global amnesia

## Abstract

**Background:**

Transient global amnesia (TGA) often shows tiny punctate hippocampal CA1 lesions on diffusion-weighted imaging (DWI). Whether these lesions represent truly transient structural alterations remains unsettled. Ultra–high-field 7-tesla (7 T) MRI increases spatial resolution and lesion conspicuity. However, the evolution of hippocampal T2 signal at 7 T from the acute phase through short-term follow-up has not been systematically examined.

**Objective:**

To determine, using serial 7 T MRI, whether hippocampal T2-weighted imaging (T2WI) abnormalities visible in acute TGA are consistently detectable acutely and completely disappear by approximately 2 weeks.

**Methods:**

Single-center consecutive case series of six patients with clinically defined TGA and DWI-confirmed CA1 lesions who underwent serial 7 T MRI, including acute imaging and follow-up at 14–18 days. The primary outcome was complete resolution of hippocampal T2 hyperintensity. Secondary outcomes were acute T2 detection rate, lesion distribution, and concordance with DWI.

**Results:**

Six patients (4 men), median age 69 years (range 50–76), all fulfilled the diagnostic criteria of TGA and no neurological deficits. Acute 7 T MRI was performed at a median of 37 h after symptom onset. Acute phase: All 6/6 showed focal hippocampal CA1 hyperintensity on 7 T T2WI, colocalizing with DWI/ADC abnormalities. Follow-up (day 15–18): T2 hyperintensity resolved in 6/6 with no visible residual signal abnormality or structural atrophy. Follow-up 7 T DWI demonstrated complete disappearance of the previously identified diffusion abnormalities in all patients. Interpretation: Even at ultra-high field, hippocampal T2 abnormalities are fully reversible within approximately 2 weeks.

**Conclusion:**

Serial 7 T MRI demonstrates that hippocampal T2WI lesions in TGA are consistently visible acutely and uniformly disappear by 2 weeks, providing high-field structural evidence that typical TGA is a transient phenomenon. These findings refine the temporal profile of hippocampal signal evolution and contribute to mechanistic understanding of CA1 vulnerability in TGA.

## Introduction

Transient global amnesia (TGA) is characterized by the abrupt onset of profound anterograde amnesia with preservation of personal identity and other neurologic functions, typically resolving within 24 h. Despite its benign clinical course, the pathophysiology of TGA remains debated. Proposed mechanisms include transient perfusion abnormalities, venous outflow impairment with Valsalva-like triggers, and network-level phenomena such as spreading-depression–like activity within vulnerable hippocampal circuits, particularly the CA1 subfield (1). Neuroimaging has been central to this discussion because it can reveal whether the syndrome leaves any structural footprint or instead represents a fully reversible disturbance.

Diffusion-weighted imaging (DWI) at conventional field strengths (1.5–3 T) frequently shows tiny punctate lesions in the hippocampal CA1 subfield in the acute to early subacute phase (2). However, DWI visibility is highly dependent on timing and sequence parameters, and negative or equivocal studies are not uncommon early after onset (3). These constraints limit the ability of DWI alone to adjudicate between competing pathophysiologic models (4). Conversely, T2-weighted imaging (T2WI) can, in principle, capture microstructural water-content changes and late tissue injury, but at clinical field strengths of routine 3 T MRI, its spatial resolution is often insufficient to reliably show the minute cortical-layer changes associated with TGA (5).

Ultra–high-field 7-tesla (7 T) MRI offers substantially increased signal-to-noise ratio and spatial resolution, improving the conspicuity of small hippocampal lesions. Prior 7 T studies have largely emphasized improved acute detection on DWI relative to lower field strengths (5, 6). By contrast, the early subacute evolution of hippocampal T2 signal at 7 T, from the acute phase through the first 2 weeks, has not been systematically characterized. This gap matters because demonstration of complete T2 normalization at ultra–high field would provide structural evidence supporting short-term reversibility in typical TGA.

We therefore conducted a consecutive, single-center observational study using serial 7 T MRI in patients with clinically defined TGA with routine 3 T DWI imaging. Our objectives were twofold: first, to determine whether acute phase 7 T T2WI consistently depicts hippocampal abnormalities that colocalize with DWI lesions; and second, to test the primary hypothesis that these T2 abnormalities uniformly resolve by approximately 2 weeks after onset. By focusing on a fixed, clinically relevant follow-up window and applying the same ultra–high-field protocol at both time points, we sought to provide a high-fidelity structural test of reversibility in typical TGA. The results are intended primarily to improve mechanistic understanding of the temporal evolution of hippocampal abnormalities in typical TGA.

This Introduction frames the study that follows, which details the consecutive cohort, standardized imaging workflow (acute and ~2-week sessions at 7 T), qualitative image assessment centered on the hippocampal CA1 subfield, and outcomes focused on T2 disappearance at follow-up.

## Methods

### Study design and participants

We conducted a single-center observational case series at Kyoto University Hospital between January 2019 and December 2023. Patients were eligible if they fulfilled established clinical diagnostic criteria for TGA according to Hodges and Warlow (7) and underwent clinically indicated 3-tesla brain MRI demonstrating a hippocampal DWI-positive lesion. Inclusion required DWI positivity on 3 T MRI. Eligible patients were invited to undergo 7 T MRI.

Six patients provided written informed consent and completed both an acute 7 T scan and a prespecified follow-up scan. The institutional Ethics Committee of Kyoto University Graduate School of Medicine approved this study (approval No. C1143), and all procedures were conducted in accordance with the Declaration of Helsinki.

### Imaging workflow and timing

All patients underwent two prespecified 7 T sessions:

Acute 7 T MRI: within 72 h of symptom onset (target window 12–48 h).

Follow-up 7 T MRI: at 14–18 days after onset.

The diagnostic 3 T scans were obtained as part of routine clinical care prior to the acute 7 T session.

### MRI acquisition

#### Clinical 3 T MRI

MRI was performed using a 3 T scanner (MAGNETOM Skyra, MAGNETOM Prisma or MAGNETOM Vida; Siemens Healthineers, Erlangen, Germany) with a 32-channel head coil or a 64-channel head/neck coil. DWI using a 2D single-shot EPI sequence with b values of 1,000 and 2,000 s/mm^2^ in 3 directions for each b value (TR/TE 5000–8,700/53–83 ms, FOV 220 × 220 mm, in-plane resolution 0.7 mm, slice thickness 3 mm with 1 mm gap, 36 slices, parallel imaging factor 2 or 3, number of excitations 2, 3 or 4. The scans with the b value of 2000 s/mm^2^ were conducted only when there was a time.

7 T MRI (MAGNETOM 7 T, Siemens Healthineers, Erlangen, Germany and a single-channel transmit volume and 32-channel receiver head coil, Nova Medical, MA, USA).

High-resolution imaging was conducted in axial and coronal to the long axis of the hippocampus for T2-weighted contrast using a 2D turbo-spin-echo sequence (TR_axial_/TR_coronal_/TE 9000/5000/65 ms, echo train length 10, FOV 176 × 164 mm, in-plane resolution 0.4 mm, slice thickness 2 mm without a gap, axial 60 slices and coronal 36 slices, parallel imaging factor 3, acquisition time 3 min 12 s) and diffusion-weighted contrast using a 2D single-shot EPI sequence with b values of 1,000 and 2,000 s/mm^2^ in 30 directions for each b value (TR/TE_b = 1,000_/TE_b = 2,000_ 4,200/55.8/63.0 ms, FOV 204 × 192 mm, in-plane resolution 1.5 mm, slice thickness 1.5 mm without a gap, 80 slices, parallel imaging factor 2, multi-band factor 2, acquisition time 2 min 42 s). The scans with the b value of 2000 s/mm^2^ were conducted only when there was a time.

#### Image handling and qualitative assessment

The analysis focused on hippocampal subfields (with emphasis on CA1 along the lateral hippocampal body).

DWI positivity was defined as a focal hyperintensity with corresponding low signal on the ADC map.

7 T T2 positivity (acute-phase) was defined as a focal, layer-like hyperintensity colocalizing with the DWI lesion.

Follow-up 7 T T2 outcome was defined as the presence or absence of residual hyperintensity at the acute lesion site. Images were qualitatively evaluated by two board-certified neuroradiologists (24 and 18 years of subspecialty practice) and one board-certified neurologist (16 years of experience). Lesion identification was based on visual assessment of DWI, ADC maps, and corresponding T2WI. For follow-up assessment, the readers evaluated the hippocampal location corresponding to the acute diffusion-restricted lesion identified on 3 T and 7 T DWI and determined whether any residual T2 hyperintensity was present at the same anatomical site. Final image interpretation was reached by consensus among the three readers. Representative images were selected and annotated with arrows to indicate lesion location. No quantitative, voxel-wise, or inter-rater agreement analyses were performed.

#### Outcomes

Primary outcome was the complete resolution of hippocampal T2 hyperintensity at two-week follow-up.

Secondary outcomes include (1) acute-phase detection rate and topographic distribution of T2 abnormalities and (2) spatial concordance between acute T2 and DWI lesions. Acute imaging was performed first, followed by prespecified follow-up imaging at 14–18 days after symptom onset.

### Statistics

Given the small sample and the qualitative objective, results were summarized descriptively without formal hypothesis testing or inter-observer agreement statistics.

## Results

Primary outcome. At two-week follow up (14–18 days), hippocampal T2 hyperintensity had completely resolved in all patients.

Secondary outcomes. In the acute phase, 7 T T2WI detected hippocampal CA1 abnormalities in 6/6, which co-localized with DWI/ADC obtained at the same time point.

### Patient characteristics

Table 1 summarizes the clinical characteristics and imaging timelines of the six patients. The median age was 69 years (range, 50–76 years), and four patients were male. The median time from symptom onset to 3 T MRI was 24 h (range, 12–42 h). Acute phase 7 T MRI was performed at a median of 37 h (range, 20–102 h); in one patient, 7 T imaging was obtained at 102 h due to scheduling constraints. Subacute 7 T MRI was obtained at a median of 15 days (range, 14–18 days). All patients demonstrated a single unilateral hippocampal lesion (right, *n* = 2; left, *n* = 4).

**Table 1:** Clinical and MRI findings in patients with transient global amnesia.

Case	Sex	Age	Onset-3 T MRI (h)	Onset-7 T MRI (h)	3 T DWI	7 T T2WI (acute)	Follow-up interval (days)	7 T T2WI (follow up)	Lesion number	Laterality
#1	M	67	12	35	Y	Y	15	Disappeared	1	Right
#2	F	57	31	102	Y	Y	14	Disappeared	1	Left
#3	M	73	18	68	Y	Y	18	Disappeared	1	Left
#4	M	76	42	20	Y	Y	14	Disappeared	1	Right
#5	M	50	37	38	Y	Y	15	Disappeared	1	Left
#6	F	71	12	35	Y	Y	15	Disappeared	1	Left

Values represent time from symptom onset to MRI acquisition. Acute 7 T MRI was prospectively targeted within 12–48 h after onset; in one patient, imaging was obtained at 102 h due to scheduling constraints. Follow up 7 T MRI was performed at 14–18 days after onset. All lesions were unilateral and confined to the hippocampal CA1 region.

MRI, magnetic resonance imaging; DWI, diffusion-weighted imaging; T2WI, T2-weighted imaging.

**Figure 1:**
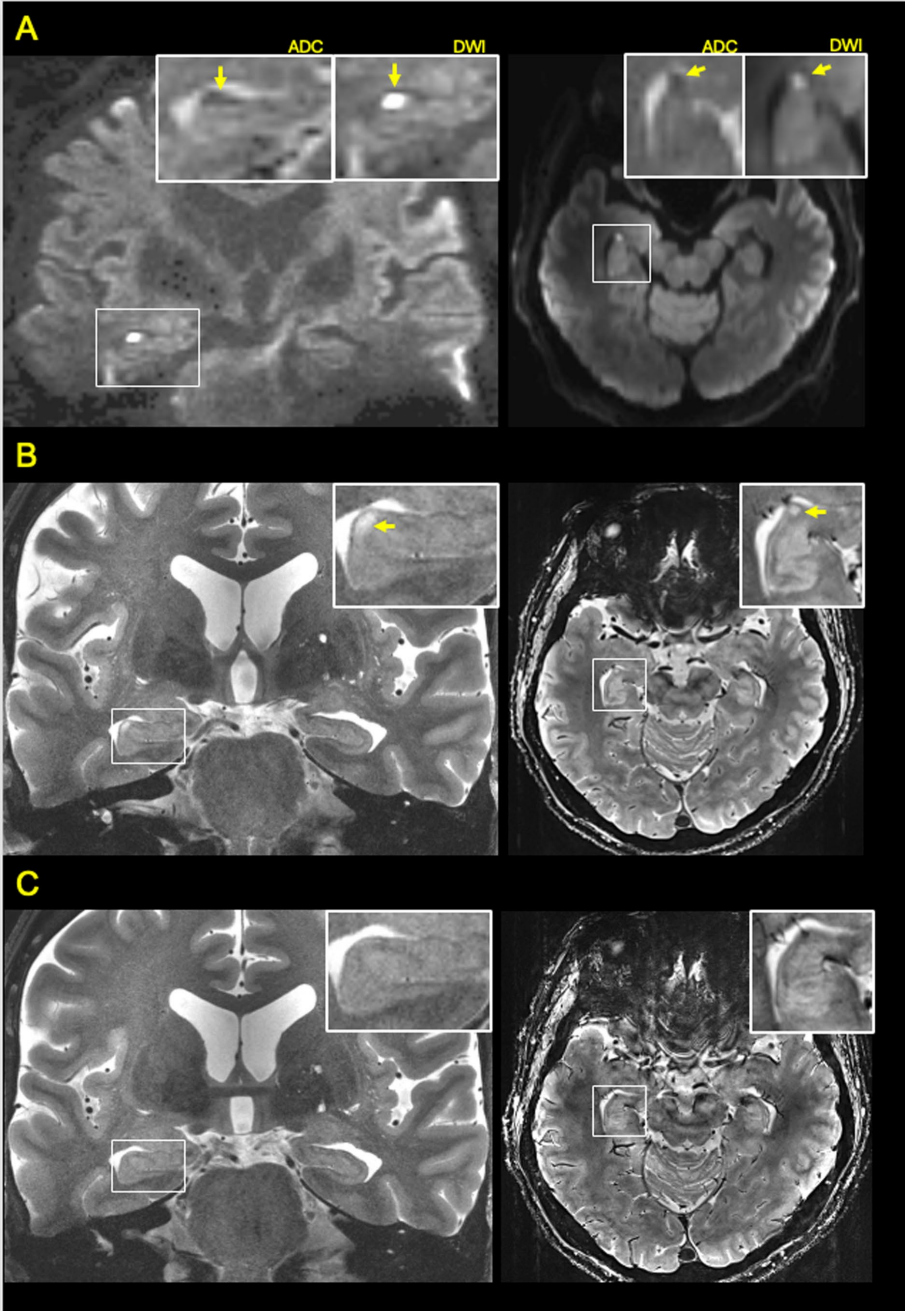
Serial 7 T MRI findings in Case 1. **(A)** Acute 7 T diffusion-weighted imaging (DWI) obtained 35 h after symptom onset demonstrates a focal punctate hyperintensity in the hippocampal CA1 region (arrow). Corresponding apparent diffusion coefficient (ADC) maps show focal hypointensity at the site of the DWI lesion, confirming restricted diffusion. Magnified views of the lesion are shown in the upper-right corner of each image; the left inset displays the ADC map, and the right inset displays the DWI image. **(B)** Corresponding 7 T T2-weighted image at the same time point shows a colocalized cortical-layer hyperintensity within CA1 (arrow), visible on both coronal and axial views. **(C)** Follow-up 7 T T2-weighted imaging performed on day 15 demonstrates complete resolution of the previously identified CA1 hyperintensity, with no residual signal abnormality.

**Figure 2:**
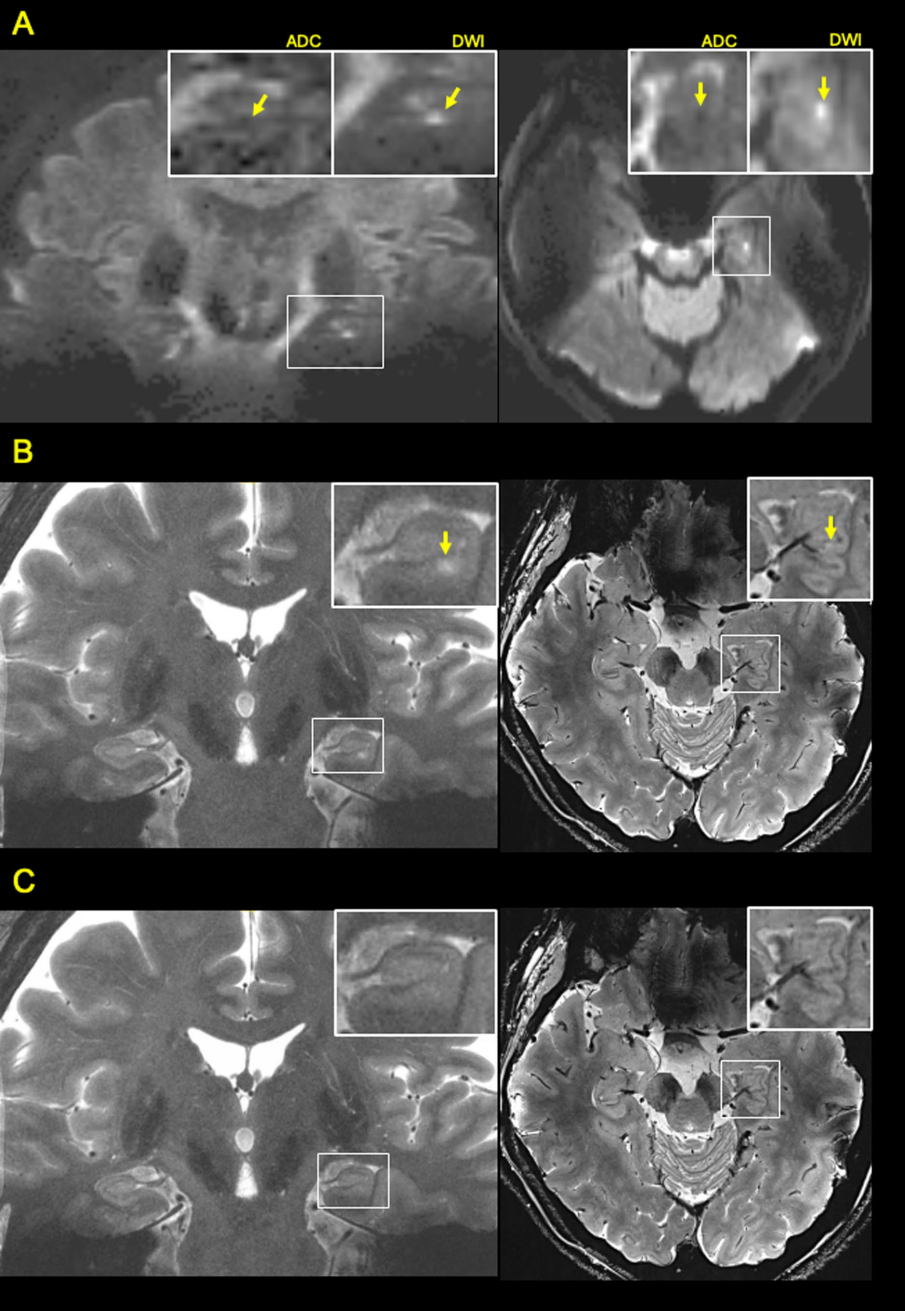
Serial 7 T MRI findings in Case 2 (delayed acute acquisition). **(A)** Acute 7 T diffusion-weighted imaging (DWI) obtained 102 h after symptom onset demonstrates a focal CA1 hyperintensity (arrow). Corresponding apparent diffusion coefficient (ADC) maps show focal hypointensity at the site of the DWI lesion, confirming restricted diffusion. Magnified views of the lesion are shown in the upper-right corner of each image; the left inset displays the ADC map, and the right inset displays the DWI image. **(B)** Concurrent 7 T T2-weighted imaging shows a corresponding cortical-layer hyperintensity within the hippocampal CA1 subfield (arrow), visible on both coronal and axial views despite the delayed acquisition. **(C)** Follow-up 7 T T2-weighted imaging performed on day 14 shows complete interval resolution of the CA1 signal abnormality.

**Figure 3:**
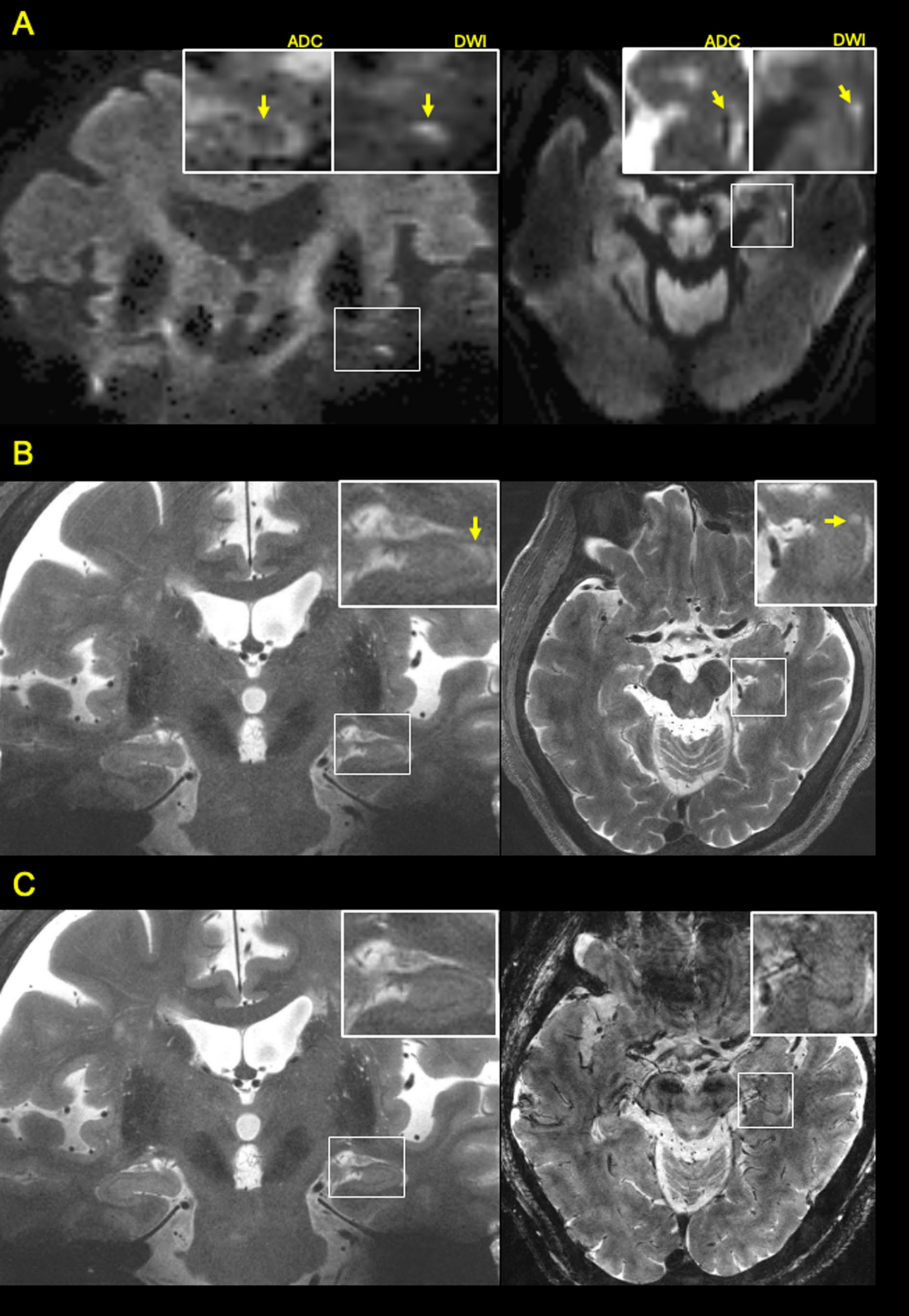
Serial 7 T MRI findings in Case 3. **(A)** Acute 7 T diffusion-weighted imaging (DWI) obtained 68 h after symptom onset demonstrates a punctate hyperintensity localized to the hippocampal CA1 region (arrow). Corresponding apparent diffusion coefficient (ADC) maps show focal hypointensity at the site of the DWI lesion, confirming restricted diffusion. Magnified views of the lesion are shown in the upper-right corner of each image; the left inset displays the ADC map, and the right inset displays the DWI image. **(B)** Corresponding 7 T T2-weighted imaging reveals a colocalized cortical-layer hyperintensity within CA1 (arrow), best appreciated on coronal reformats. **(C)** Follow-up 7 T T2-weighted imaging on day 18 demonstrates normalization of hippocampal signal with no residual hyperintensity.

**Figure 4:**
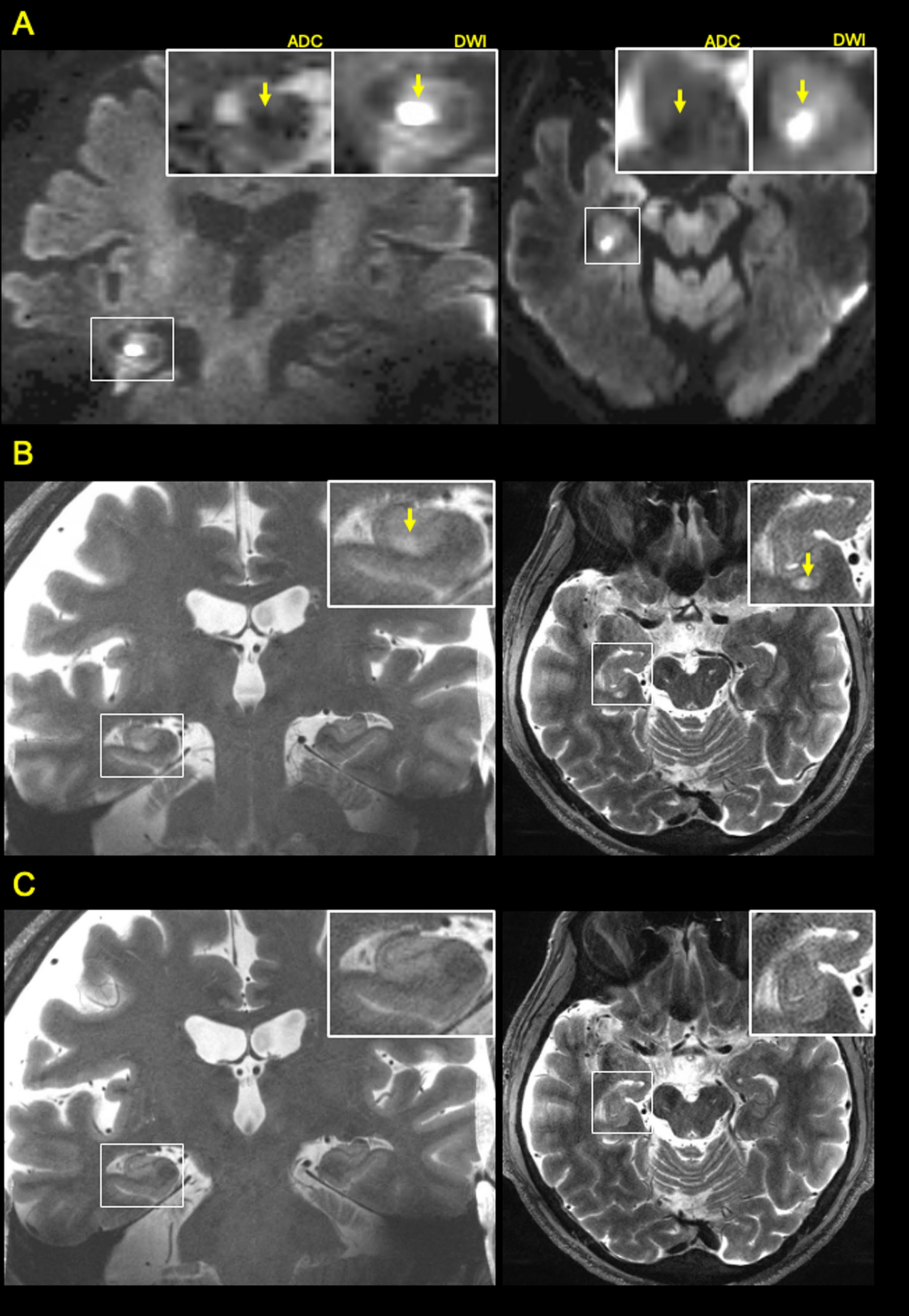
Serial 7 T MRI findings in Case 4. **(A)** Acute 7 T diffusion-weighted imaging (DWI) obtained 35 h after symptom onset demonstrates a focal CA1 hyperintensity (arrow). Corresponding apparent diffusion coefficient (ADC) maps show focal hypointensity at the site of the DWI lesion, confirming restricted diffusion. Magnified views of the lesion are shown in the upper-right corner of each image; the left inset displays the ADC map, and the right inset displays the DWI image. **(B)** Simultaneous 7 T T2-weighted imaging shows a corresponding cortical-layer signal abnormality in CA1 (arrow), evident on both coronal and axial views. **(C)** Follow-up 7 T T2-weighted imaging performed on day 15 demonstrates complete resolution of the previously observed CA1 abnormality.

Representative acute and follow-up 7 T MRI findings from Cases 1–4 are presented in Figures 1–4, whereas the corresponding findings from Cases 5 and 6 are shown in Supplementary Figures 1, 2. Follow-up 7 T DWI findings for all six patients are presented in Supplementary Figure 4.

### Acute phase (3 T clinical + 7 T research)

3 T DWI: All 6/6 patients showed a punctate hippocampal hyperintensity consistent with typical TGA lesions.

7 T DWI: The punctate lesion was detectable in all 6/6 and co-localized with the 3 T DWI abnormality.

7 T T2WI: All 6/6 patients demonstrated a corresponding cortical-layer hyperintensity localized to the hippocampal CA1 region at the acute time point. Lesions were small and confined to the hippocampus without extrahippocampal abnormalities.

Plane dependence: Lesion conspicuity varied by imaging plane. In most cases, the lesion was better appreciated on coronal reformats; however, in one case the lesion was more clearly visualized on axial images than on coronal views (Supplementary Figure 2), highlighting orientation-dependent visibility.

### Follow-up

Timing: Follow-up 7 T T2WI was obtained at day 14–18 in all patients.

Outcome: Complete resolution of the hippocampal T2 hyperintensity in 6/6 at the day 14–18 follow-up, with no residual T2 signal abnormality or structural atrophy.

DWI: Follow-up 7 T DWI was available in all six patients and demonstrated complete disappearance of the previously identified hippocampal diffusion abnormalities, with no residual focal hyperintensity (Supplementary Figure 4).

### Side and subfield

Lesions localized to the CA1 subfield of the hippocampal body/head. Laterality varied across cases; all patients demonstrated a single unilateral hippocampal lesion, and all were restricted to CA1 without extrahippocampal involvement.

## Discussion

In this consecutive six-patient series, ultra–high-field 7 T MRI revealed focal hippocampal T2WI abnormalities corresponding to abnormal signals on the DWI in the acute phase of TGA that resolved by approximately 2 weeks in all cases. To our knowledge, this is the first systematic serial 7 T report to demonstrate uniform disappearance of hippocampal T2 hyperintensity on follow-up, thereby providing structural evidence, at the highest routinely available spatial resolution, that typical TGA is structurally reversible in the early subacute phase. While prior work has largely centered on DWI (8), our findings shift the emphasis toward T2 evolution and support a model of reversible physiological disturbance rather than permanent tissue injury.

### Relationship to prior literature: from DWI sensitivity to T2 reversibility

DWI punctate lesions in the hippocampal CA1 subfield are a well-recognized hallmark of TGA, but their diagnostic yield is highly timing-dependent and sensitive to acquisition parameters (thin slices, delayed imaging at 24–96 h), with pooled meta-analytic estimates of overall detection around 40% in unselected cohorts (4). This limited and variable visibility on DWI has complicated efforts to use diffusion abnormalities alone to adjudicate competing pathophysiological theories (8). Therefore, this study specifically focused on patients with clinically typical TGA in whom focal hippocampal CA1 lesions were confirmed on routine clinical 3 T DWI prior to 7 T imaging, ensuring precise lesion localization for serial evaluation.

The longitudinal behavior of T2WI, a sequence that is theoretically sensitive to microstructural water content, gliosis, and late tissue injury, has been insufficiently characterized, particularly at 7 T where subtle residual changes would be most detectable. Our serial observations showed that acute T2 hyperintensity was consistently visible and then uniformly disappeared by 2 weeks, without any residual hyperintensity or qualitative atrophy. Thus, beyond improving acute detection (as shown for DWI), 7 T also enables a structural test of reversibility using T2 as the read-out.

### Pathophysiological implications

The DWI lesions observed in TGA are typically small, making distinction from lacunar infarction on imaging occasionally challenging. Complete normalization of T2 signal on follow-up argues against irreversible ischemic injury in typical TGA. If the acute lesions represented genuine infarction, one would expect at least minor residual T2 changes (e.g., gliosis, matrix remodeling, or subtle volume loss) in a subset of cases, especially when interrogated at 7 T. While isolated reports have described possible chronic subfield changes on ultra-high-field imaging months after TGA, these are single-case observations with heterogeneous timing and methods and remain difficult to generalize (9). Prior ultra-high-field studies have reported heterogenous findings after TGA, with some suggesting possible delayed structural alterations in CA1 (10) and others finding no persistent abnormalities on follow-up imaging (11). In contrast, our serial 7 T study prospectively tracks T2 signal from the acute phase through approximately 2 weeks and demonstrates uniform early resolution across consecutive cases, providing structural evidence for short-term reversibility. Our two-week endpoint uniformly showed no residual T2 abnormality. Consistent with these findings, follow-up 7 T DWI also demonstrated complete disappearance of the previously identified diffusion abnormalities in all patients (Supplementary Figure 4), further supporting the transient nature of hippocampal lesions in typical TGA.

Mechanistically, these results are most compatible with a reversible metabolic or perfusion-related disturbance in CA1 rather than cytotoxic injury crossing a necrotic threshold. Functional and perfusion studies have documented transient hippocampal hypoperfusion (occasionally hyperperfusion) during acute TGA with normalization on follow-up imaging (12, 13). Although perfusion imaging was not performed in the present study, future studies combining ultra-high-field structural MRI with perfusion imaging may further clarify the relationship between transient perfusion disturbances and reversible hippocampal tissue changes. Experimental work and network-level hypotheses (e.g., spreading-depression-like phenomena) likewise support transient dysfunction in vulnerable hippocampal circuits (14). Our 7 T T2 trajectory—present acutely, absent at 2 weeks—fits these models and undermines the need to invoke persistent tissue damage in typical cases.

### Venous congestion hypothesis and CA1 vulnerability

Several clinical and imaging observations point toward a venous component: TGA often follows Valsalva-like maneuvers; internal jugular vein valve incompetence and altered cervical venous drainage have been reported more frequently in TGA than in controls; and transient venous outflow impedance could produce brief, regionally selective metabolic stress in CA1, a subfield known for its exceptional susceptibility to perfusion and metabolic perturbation (15, 16). Our data neither prove nor refute venous mechanisms per se, but the absence of residual T2 change at 2 weeks—even at 7 T—suggests that any venous or perfusion disturbance remains below the injury threshold that would yield structural sequelae detectable on high-field T2WI.

### Clinical implications

These findings refine the structural time course of hippocampal CA1 abnormalities in TGA. High-resolution 7 T T2WI demonstrates that the lesion is consistently detectable acutely and uniformly resolves by 2 weeks, providing structural support for the transient nature of typical TGA. The results primarily inform mechanistic understanding rather than routine clinical decision-making, given the limited availability of ultra–high-field imaging.

### Strengths

Methodologically, this study leveraged a consecutive series with no follow-up attrition—a practical challenge in 7 T imaging—and applied a standardized protocol across acute and follow-up sessions. The consistency of the main finding across all six patients supports internal validity. Importantly, focusing on T2 evolution (rather than only DWI yield) provides new structural evidence for whether TGA is physiologically transient or structurally injurious.

### Limitations

We acknowledge several limitations. The sample size is small, reflecting both the practical barriers to serial 7 T imaging (claustrophobia, contraindications, consent) and the rarity of assembling such a complete dataset. Accordingly, this exploratory study was not powered to determine the diagnostic value or clinical justification of routine follow-up MRI in patients with transient global amnesia. Although all patients demonstrated complete resolution of T2 and DWI abnormalities, these findings should be considered hypothesis-generating and require confirmation in larger prospective studies. While a proportion of 0/6 residual T2 abnormalities has a wide upper confidence bound, the uniform direction of effect across a consecutively ascertained cohort is reassuring. Second, our follow-up window (~2 weeks) does not exclude very late microstructural signatures (e.g., iron deposition, microscopic gliosis, subtle atrophy) that might emerge over months; longer-term 7 T studies are warranted. Third, perfusion imaging was not obtained; therefore, our findings cannot directly establish the relationship between transient perfusion abnormalities and the observed reversible structural changes.

## Conclusion

Serial 7 T MRI shows that hippocampal T2 hyperintensity in TGA is consistently visible acutely and uniformly absent by 2 weeks, without residual structural change on ultra-high-field imaging. These findings provide high-resolution structural evidence that advances understanding of TGA pathophysiology. Given the limited sample size, these findings should be interpreted as preliminary and warrant validation in larger prospective studies.

## Data Availability

The original contributions presented in the study are included in the article/Supplementary material, further inquiries can be directed to the corresponding author.

## Supplementary material

SUPPLEMENTARY FIGURE 1 Serial 7 T MRI findings in Case 5. **(A)** Acute 7 T diffusion-weighted imaging (DWI) obtained 38 hours after symptom onset demonstrates a subtle focal CA1 hyperintensity (arrow), more conspicuous on axial views than on coronal reformats. Corresponding apparent diffusion coefficient (ADC) maps show focal hypointensity at the site of the DWI lesion, confirming restricted diffusion. Magnified views of the lesion are shown in the upper-right corner of each image; the left inset displays the ADC map, and the right inset displays the DWI image. **(B)** Corresponding 7 T T2-weighted imaging at the same time point shows a colocalized cortical-layer hyperintensity (arrow), better appreciated on coronal images. **(C)** Follow-up 7 T T2-weighted imaging performed on day 15 demonstrates complete resolution of the previously identified CA1 abnormality.

SUPPLEMENTARY FIGURE 2 Serial 7 T MRI findings in Case 6. **(A)** Acute 7 T diffusion-weighted imaging (DWI) obtained 35 hours after symptom onset demonstrates a focal punctate hyperintensity within the hippocampal CA1 region (arrow). Corresponding apparent diffusion coefficient (ADC) maps show focal hypointensity at the site of the DWI lesion, confirming restricted diffusion. Magnified views of the lesion are shown in the upper-right corner of each image; the left inset displays the ADC map, and the right inset displays the DWI image. **(B)** Corresponding 7 T T2-weighted imaging at the same time point shows a cortical-layer hyperintensity in CA1 (arrow), better visualized on axial views; the lesion is not clearly appreciable on coronal images. **(C)** Follow-up 7 T T2-weighted imaging performed on day 15 demonstrates interval normalization of hippocampal signal without residual abnormality.

SUPPLEMENTARY FIGURE 3 Routine clinical 3 T diffusion-weighted imaging (DWI) in Case 1–6. **(A–F)** For each case, axial images obtained prior to 7 T imaging are shown as a set of diffusion-weighted imaging (DWI), corresponding apparent diffusion coefficient (ADC) maps, and T2-weighted imaging (T2WI) arranged from top to bottom. In Case 1-2 **(A,B)**, conventional T2WI is shown, whereas in Case 3–6 **(C–F)**, T2*-weighted images were available instead of conventional T2WI. DWI demonstrates a focal punctate hyperintensity in the hippocampal CA1 region (arrow), with corresponding low signal on ADC maps, while no definite abnormality is appreciable on either T2WI or T2*-weighted images. Time from symptom onset to clinical MRI was 12, 31, 18, 42, 37, and 12 hours for Cases 1–6, respectively. These routine clinical findings established the diagnosis prior to enrollment in the serial 7 T protocol.

SUPPLEMENTARY FIGURE 4 Follow-up 7 T diffusion-weighted imaging findings in all six patients with transient global amnesia. **(A–F)** Follow-up 7 T diffusion-weighted imaging (DWI) obtained 14–18 days after symptom onset in Cases 1–6, respectively. Coronal images are shown on the left and axial images on the right. In all patients, the focal hippocampal CA1 diffusion-restricted lesion identified on acute-phase 7 T DWI had completely resolved, with no residual DWI hyperintensity. In Case 5 **(E)**, only the axial image is shown because the acute-phase lesion was not appreciable on the corresponding coronal DWI image.
